# Detection of Alpha, Beta, Gamma, and Unclassified Human Papillomaviruses in Cervical Cancer Samples From Mexican Women

**DOI:** 10.3389/fcimb.2020.00234

**Published:** 2020-06-09

**Authors:** María Guadalupe Flores-Miramontes, Dominik Olszewski, Cristina Artaza-Irigaray, Anouk Willemsen, Ignacio G. Bravo, Verónica Vallejo-Ruiz, Yelda Aurora Leal-Herrera, Patricia Piña-Sánchez, Andrea Molina-Pineda, Juan Carlos Cantón-Romero, María Guadalupe Martínez-Silva, Luis Felipe Jave-Suárez, Adriana Aguilar-Lemarroy

**Affiliations:** ^1^Centro de Investigación Biomédica de Occidente (CIBO)-Instituto Mexicano del Seguro Social (IMSS), Guadalajara, Mexico; ^2^Department of Molecular Life Sciences, University of Zurich, Zurich, Switzerland; ^3^Centre National de la Recherche Scientifique (CNRS), Laboratory MIVEGEC (UMR CNRS IRD Uni Montpellier), Montpellier, France; ^4^Centro de Investigación Biomédica de Oriente, Instituto Mexicano del Seguro Social (IMSS), Metepec, Mexico; ^5^Centro Institucional de Capacitación y Registro de Cáncer (CICyRC), Unidad Médica de Alta Especialidad (UMAE), Instituto Mexicano del Seguro Social (IMSS), Mérida, Mexico; ^6^Laboratorio de Oncología Molecular, Unidad de Investigación Médica en Enfermedades Oncológicas (UIMEO), Instituto Mexicano del Seguro Social, Mexico City, Mexico; ^7^Programa de Doctorado en Ciencias Biomédicas, Centro Universitario de Ciencias de la Salud, Universidad de Guadalajara, Guadalajara, Mexico; ^8^Unidad Médica de Alta Especialidad (UMAE), Hospital de Ginecología y Obstetricia, Centro Médico Nacional de Occidente, Instituto Mexicano del Seguro Social (IMSS), Guadalajara, Mexico; ^9^Departamento de Anatomía Patológica, Centro Médico Nacional de Occidente, Instituto Mexicano del Seguro Social (IMSS), Guadalajara, Mexico

**Keywords:** alpha, beta, gamma, HPV, cervical cancer, Mexico, papillomavirus

## Abstract

**Background:** Cervical cancer (CC) is associated to high-risk human papillomavirus (HPV) infections, for this reason it is crucial to have sensitive and accurate HPV diagnostic tests. To date, most research is focused on HPVs within the Alphapapillomavirus (α*-PVs*) genus and little attention has been paid to cervical infections with other HPV genotypes, like those of the Betapapillomavirus (β*-PVs*) and Gammapapillomavirus (γ*-PVs*) genera. The aim of this study was to determine the HPV genotypes from different genera in women with CC using Next-Generation Sequencing (NGS).

**Methods:** The study comprised 48 HPV positive CC samples evaluated with the Linear Array HPV Genotyping test and individually sequenced by 454 NGS using PGMY09/11 and FAP primers. To determine the HPV genotypes present in each sample, the obtained sequences were compared with all HPV L1 gene reference sequences from the Papillomavirus Episteme database (PaVE). Moreover, 50 HPV positive low-grade cervical lesion samples individually genotyped with NGS were also included to determine the genotypes present preferentially in CC patients.

**Results:** Among the 48 CC samples, 68.75% consisted of multiple HPV infections, 51 different genotypes were detected, of which 7 are still unclassified, 28 belong to α*-PVs* (6, 11, 16, 18, 26, 30, 33, 35, 39, 42, 43, 44, 45, 51, 52, 53, 54, 59, 62, 66, 68, 69, 70, 71, 74, 81, 102, 114), 10 to β*-PVs* (5, 12, 21, 37, 38b, 47, 80, 107, 118, 122), and 6 to γ*-PVs* (101, 103, 123, 135, 147, 214). Among them, HPV16 was the most prevalent genotype (54.2%), followed by HPV18 (16.7%), HPV38b (14.6%), and HPVs 52/62/80 (8.3%). Some genotypes were exclusively found in CC when compared with Cervical Intraepithelial Neoplasia grade 1 (CIN1) samples, such as HPVs 5, 18, 38b, 107, 122, FA39, FA116, mSK_120, and mSK_136.

**Conclusions:** This work demonstrates the great diversity of HPV genotypes detected by combining PGMY and FAP primers with NGS in cervical swabs. The relatively high attribution of β*-* and γ*-* PVs in CC samples suggest their possible role as carcinogenic cofactors, but deeper studies need to be performed to determine if they have transforming properties and the significance of HPV-coinfections.

## Introduction

Human Papillomaviruses (HPVs) are small circular double-stranded DNA viruses that infect mucosal or cutaneous epithelium and that in most of the cases persist asymptomatically during the host's lifetime. However, certain HPV infections can cause clinical manifestations that range from benign lesions to malignant tumors such as skin or cervical cancers (Bravo and Félez-Sánchez, 2015). HPVs typically contain 8 genes in their 8 kb genome, including the *L1* gene that encodes the major capsid protein and is used for virus classification as its nucleotide sequence is well-conserved among HPVs. A papillomavirus genotype is a full-length genome whose *L1* gene sequence differs in at least 10% from that of any other papillomavirus (Bernard et al., 2010). HPVs are grouped in five major phylogenetic genera referred to as *Alpha (*α*)*-, *Beta(*β*)-, Gamma(*γ*)*-, *Mu(*μ*)*-, and *Nu(*ν*)*- *papillomaviruses*, where viruses that belong to different genera have <60% similarity within the *L1* gene. Within a genus, HPVs are classified in species that share between 60 and 70% of similarity (Bzhalava et al., 2015). According to the International HPV Reference Center (Karolinska Institute), 226 HPV genotypes have been described to date, but new HPVs are continuously being found^1^. It is worth noting that the number of HPVs belonging to the β*-* and γ*- PV* genera has rapidly increased in the last years (Mühr et al., 2018). Concerning the α*-PV* genus, it contains the carcinogenic HPVs called high-risk (hr-HPVs). If those viruses persist over time in their site of infection, they can cause cervical cancer (CC), one of the most common cancers in women worldwide, as well as cancers of vagina, vulva, penis, anus, and oropharynx (Schiffman et al., 2016). HPV16 is the predominant genotype causing invasive CC and other HPV-related cancers worldwide (Guan et al., 2012). Therefore, to efficiently detect HPV16 and the other genotypes, HPV screening programs in reach of most of the target-population must be implemented. Apart from primary prevention with HPV vaccination, screening for cervical lesions using cytology and HPV tests is a necessary approach (Bosch et al., 2016). Even though the great majority of α*-PVs* infect mucosa in the anogenital epithelia, some viral genotypes from this genus were originally described as cutaneous, together with β*-* and γ*-PVs*. However, regarding β*-PVs*, it has been difficult to determine their individual role in cutaneous malignancies due to their high diversity and their dissemination throughout many tissues such as the skin, the oral cavity, the nasal mucosa, and the anogenital region (Nunes et al., 2018).

In recent years, the development of highly sensitive HPV detection methods has allowed the identification of a large number of new HPVs and their ubiquity in samples from different origin. For research purposes, sensitive, and specific technologies that cover a broad range of HPV genotypes have been developed (Nilyanimit et al., 2018). More precisely, the use of PGMY and FAP primers couple with Next-Generation Sequencing (NGS) strategies detect α-, β-, and γ*-PVs* simultaneously in different tissues have helped to broaden the knowledge in this field (Flores-Miramontes et al., 2015; Sias et al., 2019). The aim of this work was to study the HPV genotypes present in cervical samples from women diagnosed specifically with CC using NGS with primer pairs (both PGMY11/09 and FAP) that allow amplification not only of α*-PVs*, but also of multiple β- and γ*-PVs*.

## Materials and Methods

### Sample Collection

In this study, more than 50 CC samples were collected from women who attended a medical examination at the Hospital de Ginecología y Obstetricia, Centro Médico Nacional de Occidente, IMSS in Guadalajara, Jalisco, Mexico. Additionally, more than 100 CIN1 (Cervical Intraepithelial Neoplasia grade 1) samples were collected both in the hospital mentioned above, as in the Hospital General de Zona # 12, Lic. Benito Juárez, IMSS, in Mérida, Yucatán. Sample recruitment was done by gynecologists throughout 2016–2017, by collecting the cervical swabs in Preserv-Cyt medium solution (Cat. no. 70097-002, Hologic, Inc., Marlborough, MA, USA). All diagnoses were confirmed by immunohistopathological analysis. It is meaningful to mention that the samples were collected only from women who had not previously had any type of treatment.

This study was approved by the Ethics and Research Committees of the Instituto Mexicano del Seguro Social (IMSS), with registration number R-2014-785-036. All women provided written informed consent before the sample collection.

### DNA Extraction and Linear Array HPV Genotyping Test

Total DNA was extracted from each cervical sample using the AmpliLute Liquid Media Extraction Amplicor kit (Cat. no. 03750540190; Roche Molecular Systems, Inc., Branchburg, NJ, USA.). HPV positivity was determined by the *Linear Array HPV Genotyping Test* (Cat. no. 03378179190, Roche Molecular Systems) according to the manufacturer's instructions. Two CC samples and six CIN1 samples were excluded due to low DNA concentrations.

### HPV Genotyping by 454 Next-Generation Sequencing

Considering the positivity to HPV by *Linear Array*, 48 CC samples were selected to perform NGS. Additionally, two groups of CIN1 samples were chosen: (1) a group of 23 samples in which each sample had coinfection with at least 3 genotypes (named “multiple-infection group”) and (2) a group of 27 samples that were HPV negative by *Linear Array*, but positive by FAP primers amplification (named “PGMY-HPV-neg group”).

First, the DNA of each sample was amplified by two independent conventional PCRs with PGMY09/11 primers on one side and FAP59/64 primers on the other side (Forslund et al., 1999; Gravitt et al., 2000). Then, a second PCR reaction was performed using PGMY09/11 on one side and FAP6085/64 primer pairs on the other side, coupled to a universal tail sequence as previously described (Flores-Miramontes et al., 2015). PCR products were quantified using the Qubit dsDNA HS (Cat. no. Q32854, Life Technologies, Eugene, OR, USA), diluted to 5 × 10^9^ molecules per microliter and 454 MIDs from Multiplicom NV CFTR kits individual barcodes were added to each sample (Cat. nos. ML-0008.192, ML-0016.192, and ML-0124.192, Molecular Diagnostics, Niel, Belgium). Afterward, samples were purified with the Agentcourt AMPure XP beads (Cat. no. A63880, Beckman Coulter Genomics, Danvers, MA, USA) and evaluated for their quality with the Agilent DNA 1000 kit (Cat. no. 5067-1504, Agilent Technologies, Santa Clara, CA, USA) on the 2100 Bionalyzer (Cat. no. G2939BA, Agilent Technologies). Finally, 454 NGS was performed using GS Junior Titanium kits in a GS Junior Sequencer, following the manufacturer's instructions (Cat. no. 05996562001, 05996589001, 05996597001, and 05996619001, Roche Diagnostics, Basel, Switzerland).

### Data Analysis

The sequencing data were first analyzed with the FastQC tool to determine the quality of the sequences^2^. Then, to identify specific HPV genotype sequences present in each cervical sample, the *Roche GS Reference Mapper v3.0* software was used, taking as references all the *L1* sequences reported in the Papillomavirus Episteme (PaVE) database (Van Doorslaer et al., 2012). The parameters considered for mapping were the following: trimming of adapters and primers, 95% of minimum overlap identity, Phred score ≥20, minimum read length of 150 bp and exclusion of all the repeated reads.

Additionally, to compare the results obtained with the *Roche GS Reference Mapper v3.0* software, the following pipeline was performed: (1) the *AlienTrimer v. 0.4.0* software was used to remove all adapter sequences and specific primers (Criscuolo and Brisse, 2013), (2) *Printseq v. 0.20.4* software was utilized to filter all sequences with a *Phred Score* >20, remove sequences <150 nucleotides, and trim 12 additional nucleotides from the 5′ end (Schmieder and Edwards, 2011), (3) to mapping all reads both Bowtie 2 (Langmead and Salzberg, 2012) and Megablast (Zhang et al., 2000; Morgulis et al., 2008) were used.

All HPV raw sequence reads were deposited in the NCBI BioProject repository with the following accession numbers: PRJNA506700 (for CC samples), PRJNA506685 (for CIN1 samples with multiple HPV infections), and PRJNA506459 (for PGMY-HPV-negative CIN1 samples). HPVs that appeared in a sample with only 1 read and <150 bp or sequences that aligned to an HPV genotype with <95% of identity (checked with Nucleotide BLAST) were excluded.

### HPV Genotype Prevalence and Attribution

The prevalence of the different HPV genotypes that are present in the CC samples under study is calculated by dividing the total number of type-specific positive samples by the total number of samples (in this work, 48 samples that are HPV positive). However, when multiple HPV infections are present, as it is the case in this study, the prevalence could probably over- or under- estimate the attribution of individual HPV genotypes. The contribution of each HPV genotype in multiple infections to lesion development is being investigated, but it is likely that some HPVs play a more dominant role than others (Insinga et al., 2008). Therefore, the relative attribution of each HPV was estimated in the 48 CC samples.

To calculate the relative attribution based on the presence of one or more HPVs, a value of 1 was assigned to samples with single infection, while in coinfections, a proportional fraction value (based on the number of HPVs detected in the same sample) was assigned. For example: assuming that in one sample there are 4 HPVs, a value of 0.25 was assigned to each HPV genotype. The global relative attribution of each HPV was obtained by adding all these values assigned to that specific HPV in all samples and dividing it by the total number of samples that amplified a sequence with PGMY (*n* = 41) or FAP (*n* = 39).

### HPV Tree Construction

Two different analysis were carried out using the MEGA X software (Kumar et al., 2018), one including only the 41 HPV consensus sequences amplified with FAP primers (≈350 nucleotides) and the other containing the 19 HPV consensus sequences amplified with PGMY (≈450 nucleotides). The analyses were performed separately because the set of primers binds in different L1 positions. The sequences were aligned using the MUSCLE algorithm (Edgar, 2004), then the Maximum likelihood Statistical method and partial deletion was chosen to construct the phylogenetic tree. The radial form was selected.

## Results

### *Alpha-, Beta-*, and *Gamma- Papillomaviruses* Are Detected With NGS in Cervical Cancer Samples

Of the 48 CC samples sequenced with 454 NGS, 33 samples amplified HPVs with both set of primers (FAP and PGMY), 9 only with PGMY and 6 only with FAP. A total of 51 different HPV genotypes were detected, 7 of them are yet unclassified and had more than 95% identity with the following GenBank sequences: FA39 (AF217684.1), FA116 (AY468411.1), HPV-mSK_120 (MH777262.2), HPV-mSK_136 (MH777278.1), HPV-mSK_220 (MH777359.2), HPV-mSK_221 (MH777360.2)/EV03c40 (MF588746.1), and w11C24 (MF588709.1)/HPV-mSK_036 (MH777184.1). The other 44 HPVs are classified in different genera: 28 α*-PVs* (6, 11, 16, 18, 26, 30, 33, 35, 39, 42, 43, 44, 45, 51, 52, 53, 54, 59, 62, 66, 68, 69, 70, 71, 74, 81, 102, and 114), 10 β*-PVs* (5, 12, 21, 37, 38b, 47, 80, 107, 118, and 122), and finally 6 γ*-PVs* (101, 103, 123, 135, 147, and 214). A detailed description of the HPVs genotypes detected in each sample are described in Table 1, including the number of reads detected with PGMY or FAP primers, and the genotypes detected with the *Linear Array HPV Genotyping test*.

**Table 1:** HPV genotypes found in each of the 48 cervical cancer samples.

**Sample code/Diagnosis**	**HPV type**	**Genotypes included in LA**	**Positive HPVs detected by LA**	**Positive HPVs detected by NGS**	**Reads by NGS**	
					**FAP**	**PGMY**
S10M1	16	^*^	^*^	^*^	9	481
ISCC	18	^*^	^*^	–	–	–
	62	^*^	^*^	^*^	892	3
S10M2	12	–	–	^*^	21	0
ISCC	21	–	–	^*^	1,067	0
	33	^*^	^*^	^*^	1	507
	62	^*^	–	^*^	3	0
S10M3	16	^*^	^*^	^*^	233	377
SCC	38b	–	–	^*^	1,002	0
	123	–	–	^*^	25	0
S10M4	6	^*^	–	^*^	269	0
ISCC	11	^*^	–	^*^	416	0
	30	–	–	^*^	499	0
	66	^*^	^*^	^*^	0	284
S10M5	43	–	–	^*^	2	0
ISCC	69	^*^	^*^	^*^	0	49
	71	^*^	^*^	^*^	0	94
	81	^*^	^*^	^*^	979	225
	84	^*^	^*^	–	–	–
	114	–	–	^*^	0	3
S10M6	16	^*^	–	^*^	0	50
SCC	39	^*^	^*^	^*^	0	2
	62	^*^	^*^	^*^	1,079	218
	71	^*^	^*^	^*^	0	100
S10M7	44	–	–	^*^	16	0
SCC	52	^*^	^*^	^*^	210	342
	53	^*^	^*^	–	–	–
	68	^*^	^*^	–	–	–
	80	–	–	^*^	767	0
S10M8	16	^*^	^*^	^*^	0	174
SCC	54	^*^	^*^	^*^	762	3
	62	^*^	^*^	^*^	105	0
	70	^*^	^*^	^*^	164	347
	118	–	–	^*^	2	0
	HPV–mSK_036/w11C24	–	–	^*^	67	0
S10M9	35	^*^	^*^	^*^	0	450
ISCC	38b	–	–	^*^	735	0
S10M10	16	^*^	^*^	^*^	350	484
IADC	38b	–	–	^*^	401	0
	47	–	–	^*^	109	0
S10M11	16	^*^	^*^	^*^	148	435
ISCC	38b	–	–	^*^	890	0
	52	^*^	^*^	–	–	–
S10M12	16	^*^	^*^	^*^	115	160
SSC	52	^*^	^*^	–	–	–
	74	–	–	^*^	330	0
S10M13	16	^*^	–	^*^	143	431
ISCC	33	^*^	^*^	^*^	183	514
	38b	–	–	^*^	1,377	0
S10M14	16	^*^	^*^	^*^	0	583
ISCC	107	–	–	^*^	309	0
S10M15	16	^*^	^*^	^*^	0	360
ISCC	HPV–mSk_221/EV03c40	–	–	^*^	3	0
	FA39	–	–	^*^	1,158	0
S10M16	16	^*^	^*^	^*^	1	370
ISCC	42	^*^	–	^*^	950	0
S10M17	16	^*^	^*^	^*^	0	306
ISCC	122	–	–	^*^	502	0
	135	–	–	^*^	550	0
S10M18	5	–	–	^*^	747	0
ISCC	16	^*^	–	^*^	0	3
	45	^*^	^*^	^*^	0	261
	66	^*^	–	^*^	0	19
	80	–	–	^*^	2	0
S10M19	16	^*^	^*^	^*^	0	335
ISCC	HPV–mSK_120	–	–	^*^	339	0
S10M20	16	^*^	^*^	^*^	13	410
SCC	38b	–	–	^*^	627	0
S10M21	18	^*^	–	^*^	8	0
ISCC	42	^*^	–	^*^	1,362	0
	45	–	^*^	–	–	–
S10M22	31	^*^	^*^	–	–	–
ISCC	54	^*^	^*^	–	–	–
	71	^*^	^*^	–	–	–
	83	^*^	^*^	–	–	–
	89	^*^	^*^	–	–	–
	101	–	–	^*^	2,092	0
	102	–	–	^*^	115	0
	214	–	–	^*^	6	0
S10M23	18	^*^	^*^	–	–	–
IADC	52	^*^	^*^	–	–	–
	80	–	–	^*^	2,188	0
S10M24	71	^*^	^*^	–	–	–
SCC	80	–	–	^*^	1,914	0
S11M1	16	^*^	^*^	^*^	151	285
SCC	52	^*^	–	^*^	3	0
S11M2	16	^*^	–	^*^	2	0
SCC	52	^*^	^*^	^*^	2,369	418
	53	^*^	^*^	^*^	1	0
	66	^*^	^*^	–	–	–
	68	^*^	^*^	^*^	0	1
S11M3	51	^*^	^*^	^*^	0	231
ISCC	HPV–mSK_136	–	–	^*^	100	0
S11M4	16	^*^	^*^	^*^	158	302
IADC	18	^*^	^*^	^*^	4	1
	30	–	–	^*^	514	0
	38b	–	–	^*^	140	0
S11M5 ISCC	16	^*^	^*^	^*^	225	335
S11M6 IADC	59	^*^	^*^	^*^	558	461
S11M7 ISCC	16	^*^	^*^	^*^	124	412
S11M8 ADC	16	^*^	^*^	^*^	210	223
S11M9	18	^*^	^*^	^*^	3	0
ISCC	26	^*^	^*^	^*^	371	140
S11M10	107	–	–	^*^	846	0
ISCC	FA39	–	–	^*^	137	0
	HPV–mSK_220	–	–	^*^	7	0
S11M11	16	^*^	^*^	–	–	–
SCC	18	^*^	^*^	^*^	85	0
	FA116	–	–	^*^	1,381	0
S11M12	18	^*^	^*^	^*^	3	1
ADC	37	–	–	^*^	246	0
	147	–	–	^*^	269	0
S11M13	16	^*^	^*^	^*^	4	0
ISCC	18	^*^	–	^*^	83	1
	122	–	–	^*^	932	0
	FA116	–	–	^*^	1,655	0
S11M14	5	–	–	^*^	597	0
ADC	45	^*^	^*^	–	–	–
	103	–	–	^*^	681	0
S11M15 ISCC	16	^*^	^*^	^*^	0	707
S11M16 ISCC	16	^*^	^*^	^*^	0	581
S11M17	16	^*^	^*^	–	–	–
ISCC	59	^*^	^*^	^*^	0	671
S11M18	39	^*^	^*^	–	–	–
ISCC	68	^*^	^*^	^*^	0	1,235
S11M19	16	^*^	^*^	^*^	0	503
ISCC	52	^*^	^*^	–	–	–
S11M20	39	^*^	^*^	^*^	0	983
ISCC	52	^*^	–	^*^	5	0
	71	^*^	^*^	–	–	–
S11M21 IADC	33	^*^	^*^	^*^	0	771
S11M22 ADC	18	^*^	^*^	^*^	0	892
S11M23 IADC	18	^*^	^*^	^*^	0	779
S11M24 ISCC	16	^*^	^*^	^*^	0	553

*Genotyping was performed either by Linear Array Genotyping Test or by 454 NGS using FAP or PGMY primer sets. The number of reads is also included*.

* *Show presence and (–) absence. SCC, Squamous Cell Carcinoma; ISCC, Invasive Squamous Cell Carcinoma; ADC, Adenocarcinoma; IADC, Invasive Adenocarcinoma. HPV genotypes that have been classified by the IARC as high and probable risk for humans are shown in red*.

**Figure 1:**
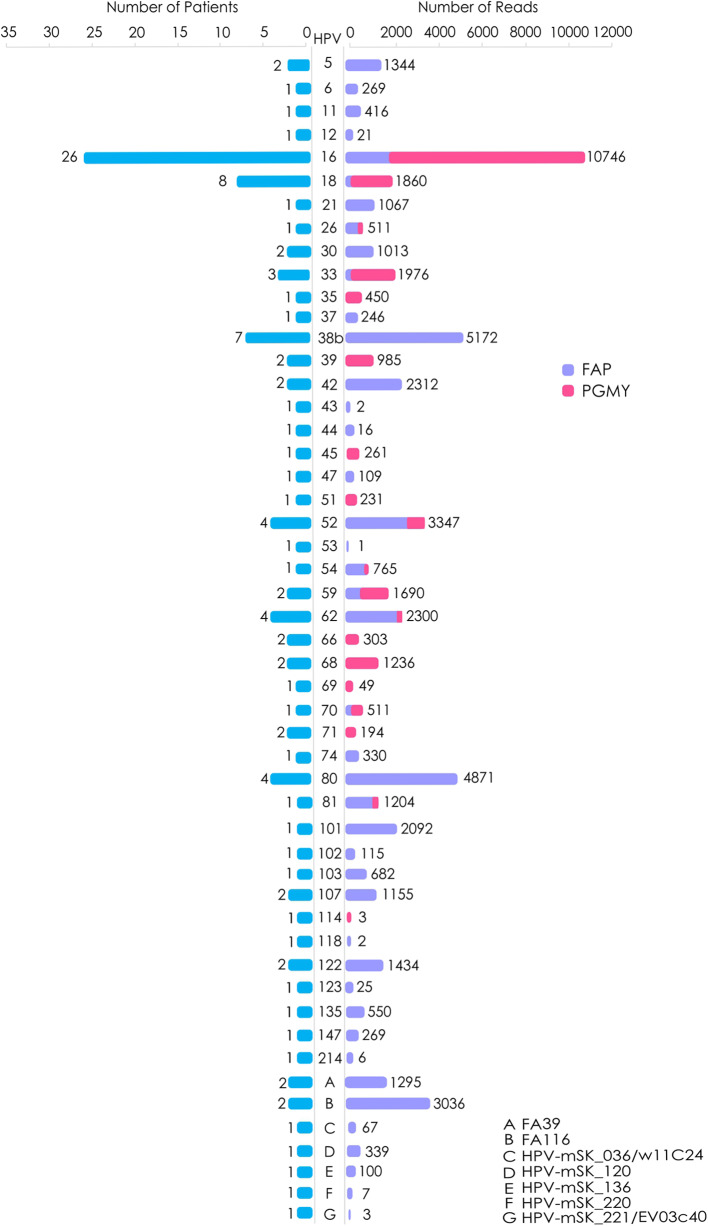
Frequency of the HPV genotypes found with NGS in CC samples. The graphic shows the number of patients positive to each specific HPV genotype as follow: (blue) **(Left)** and the total of reads given for each HPV by NGS, either with PGMY (pink) or FAP primers (purple) **(Right)**. A–G: HPV genotypes not yet classified.

To summarize the data, our findings are schematized in Figure 1, in which the global number of reads from each HPV genotype are depicted, including the number of patients in which each genotype was found. The HPV genotypes with the greatest number of total reads were: 16 (with 10746 reads), 38b (5172), 80 (4871), 52 (33047), and FA116 (3036).

### HPV Prevalence by Using PGMY or FAP Primers Coupled With NGS

**Figure 2:**
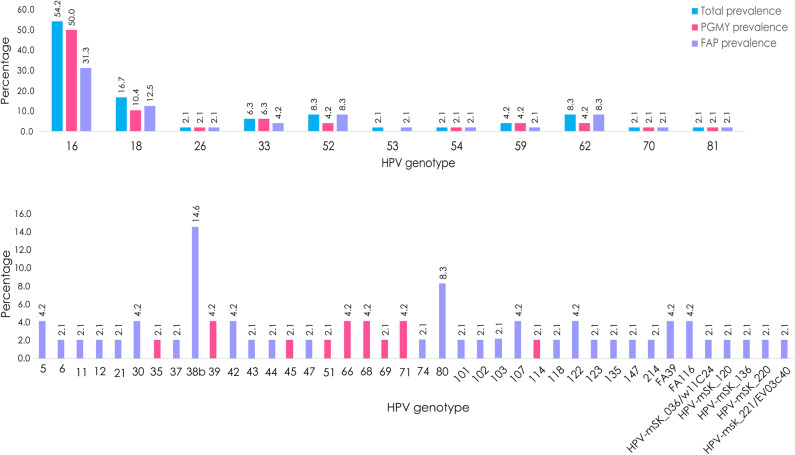
HPV prevalence by using NGS determined in CC samples. Percentage of total HPV type-specific prevalence (blue), prevalence observed by using PGMY primers (pink), or FAP primers (purple).

The global prevalence of α-, β-, γ-, and unclassified- PVs (considering coinfections in the 48 samples) was 93.8, 39.6, 10.4, and 14.6%, respectively. Interestingly, considering only the NGS data, 68.75% (*n* = 33) of the samples had multiple HPV infections. The most prevalent genotype found was HPV 16 (*n* = 26, 54.2%), followed by 18 (*n* = 8, 16.7%), 38b (*n* = 7, 14.6%), 52/62/80 (*n* = 4, 8.3%), and 33 (*n* = 3, 6.3). It is important to mention that, as visualized in Figure 2, HPV 38b and 80 could be detected exclusively with the FAP primers, and that HPVs 18, 52, and 62 were detected in some samples only by using FAP primers.

Of the 51 HPV genotypes detected, only 11 (all of them from the α-genus), were amplified with both PGMY and FAP primer sets: 16, 18, 26, 33, 52, 53, 54, 59, 62, 70, and 81, while 31 were exclusively amplified with FAP and 9 with PGMY (Figure 2). It should be noted that the HPVs most frequently detected with FAP primers were: 38b, 80, and 5/30/42/107/122/FA116/FA39.

### Relative Attribution of Each HPV Genotype in the Cervical Cancer Samples

**Figure 3:**
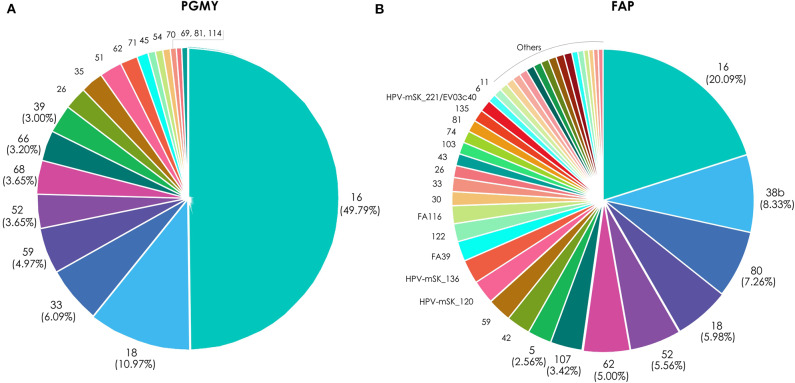
Relative attribution based on HPV type-specific presence in CC samples. The graphics depict the attribution percentages of each HPV found in the 48 cervical cancer samples, either amplified with the PGMY primers **(A)** or with FAP primers **(B)**.

For a more precise analysis, due to the high HPV genotype coinfection rates, type-specific HPV attribution to CC was calculated (as detail explained in materials and methods), based on their amplification either with PGMY or FAP primers (Figure 3). Taking into consideration the HPV genotypes that amplified with PGMY primers, those with the higher relative attribution were: HPV 16 (49.8%), 18 (11.0%), 33 (6.1%), 59 (4.9%), 52/68 (3.7%), 66 (3.3%), 39 (3.0%), and 26/35/51 (2.4%); whereas with FAP primers the HPVs with higher attribution were: 16 (20.1%), 38b (8.3%), 80 (7.3%), 18 (6.0%), 52 (5.6%), 62 (5.0%), 107 (3.4%), and 5/42/59/HPV-mSK_120/HPV-mSK_136 (2.6%).

### Unclassified HPV Genotypes

**Figure 4:**
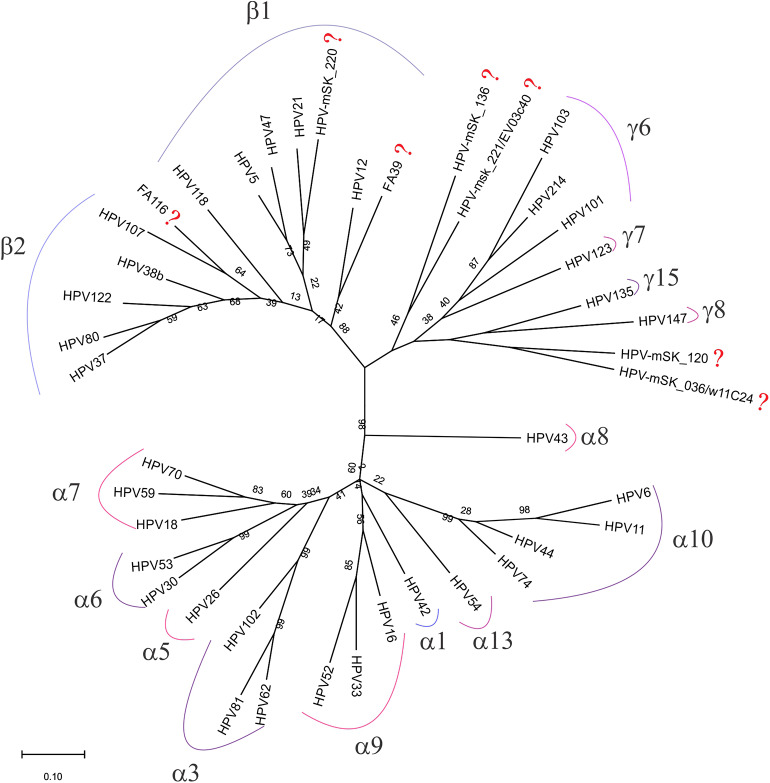
Phylogenetic tree built with the 42 HPV consensus sequences obtained only by FAP primers amplification in CC samples. Analysis was performed by MEGA X software as described in materials and methods; the genera and species from each group are included. The symbol (?) shows all those sequences still unclassified.

To predict to which genus belong each one of the seven unclassified HPV genotypes detected in the CC samples, a phylogenetic analysis was performed using all the consensus sequences obtained by NGS (as described in the materials and methods section). As visualized in Figure 4, three of the seven genotypes were found to be very closely related to β2 and β1 species (HPV-FA116, HPV-mSK_220, and HPV-FA39), while HPV-mSK_136, HPV-mSK_221/EV03c40, HPV-mSK_220, and HPV-mSK_036/ w11C24 were found to belong to the γ- genera.

### Comparison of *Alpha-, Beta-*, and *Gamma- Papillomaviruses* Detected With NGS in Low-Grade Cervical Lesions

**Figure 5:**
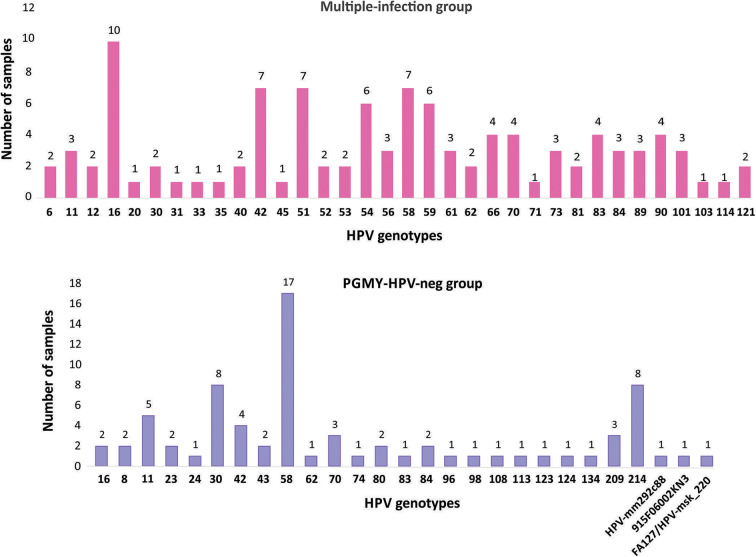
Frequency of the HPV genotypes found with NGS in CIN1 samples. The samples are divided into two groups: the “Multiple-infection group” and the “PGMY-HPV-neg group.” In the first group, NGS was performed from amplicons using the two sets of primers, PGMY and FAP; in the 2nd group they were only amplified with FAP primers.

To contrast the HPV genotypes that are circulating in CC by using NGS, samples of low-grade cervical lesions (exclusively CIN1 lesions) were also individually sequenced. Firstly, we genotyped the 100 samples using the *Linear Array Genotyping test* and 59% tested positive for HPV. Of these, we selected 23 samples that were positive for at least three genotypes (named “Multiple-infection group”) and 27 samples negative to HPV through the same test, but positive when using FAP primers (named PGMY-HPV-neg group). As depicted in the graphs of Figure 5, the most frequent genotypes found in the first group were: 16, 42/51/58/, 54/59, and 66/70/83/90. Interestingly, in the PGMY-HPV-neg group, the most frequent genotype was 58 (found in 17 out of the 27 samples), followed by 30/214 (8/27 each), 11 (5/27), 42 (4/27), and 70/209 (3/27 each). Additionally, 3 unclassified HPV genotypes were found: HPV-mm292c88 (MF588683.1), HPV-MSK_220 (MH777359.2), and the HPV isolate 915 F 06 002 KN3 (JF966374.1).

## Discussion

Accurate HPV genotyping is necessary for an effective screening of the cervical injuries and for epidemiological studies. Although there is already a wide overview of the circulating HPVs worldwide, detection methods have been focused only on α*-PVs*. In order to understand which HPVs infect the cervical mucosa of Mexican women that have developed CC, HPV genotyping was performed in 48 CC samples by using different primer pairs (PGMY09/11 and FAP6085/64) that amplified not only α- but also β and γ*-PVs*, followed by Next-Generation Sequencing. Additionally, CIN1 samples were also included to contrast the HPV genotypes that are circulating in both pathological groups.

This study contributes to the crescent evidence that β*-* and γ*- PVs* are also present in CC samples. Recently, in a study performed in cervical samples among asymptomatic women from Brazil (Ludwig/McGill Cohort Study), Sichero et al. determined β*-PVs* by a type-specific, multiplex genotyping PCR assay (amplifying E7 gene), followed by HPV genotyping using a bead-based Luminex technology; and reported that β*-PV* infections were more frequent than those observed with α*-PVs*, however, authors assume that detection of β*-PVs* in the cervix may be unrelated to an active infectious process (Sichero et al., 2017). Whether β-, or γ- *PVs* could have a relevant role as cofactors in the carcinogenic progression or a first infection with high-risk α*-PVs* is needed, are questions that need to be addressed, as further discussed.

In our study, in CC samples, a high percentage of β-, γ-, and unclassified *PV*s genera were found (36.9, 10.4, and 14.6%, respectively). Additionally, using NGS, a great proportion of coinfections were observed (68.8%). In comparison, when the same 48 samples were genotyped with the Linear Array HPV Genotyping Test, only 35.4% were found in coinfections (Table 1). This data present a similar trend to what was previously found by our research group, in which, by using the Linear Array HPV Genotyping test, only 26% of the CC samples showed coinfections (Aguilar-Lemarroy et al., 2015). Some other studies in which NGS has not been used, have also reported a low rate of coinfections in CC samples, as described by Senapaty et al., who found 22.67% of coinfections in 172 CC cases (Senapati et al., 2017), or as described by Pimenoff et al., where they analyzed 8780 HPV DNA-positive invasive CC cases worldwide and found that 6.7% contained multiple HPVs (Pimenoff et al., 2019). These contrasting differences show that most of the single infections detected with commercially tests (such as the Linear Array HPV Genotyping Test) are indeed multiple infections that should be evaluated with a more precise HPV detection method.

Concerning the coinfection rates in this study, we observe that 33.3% (*n* = 11) were multiple infections with α*-PVs* exclusively, while 60.6% (*n* = 20) were α*-PVs* in coinfection with 1 or more β-, γ-, or unclassified- *PVs*, whereas 6.1% (*n* = 2) samples lack of any α*-PV*. Recent studies have also observed coinfections with HPVs from α-, β-, and γ- genera in head and neck cancer (Agalliu et al., 2016; Sabol et al., 2016).

Regarding the prevalence in CC samples, HPV 16 was the most common detected genotype (26 out of 48) found in 7 cases (27%) as a single infection, and in 19 cases (73%) as a multiple infection. HPV16 had the higher prevalence and attribution, as reported worldwide by many authors and particularly in a study comprising 8977 HPV positive invasive CC samples from 38 countries in Europe, North America, central South America, Africa, Asia, and Oceania (de Sanjose et al., 2010). We found this genotype mainly together with HPV 38b (*n* = 6), HPV 62 (*n* = 3), and HPVs 18/52/122 (*n* = 2). In addition, we also determined that HPV 38b and 80 are common in the Mexican population with CC, interestingly, both genotypes belong to β2 species. Until now, β*-PVs* are considered mainly to have a cutaneous tropism even if they have been detected in other anatomical sites apart from the skin, and they are suspected to promote non-melanoma skin cancer development together with ultraviolet radiation (Tommasino, 2017).

Particularly, HPV 38 E6 and E7 were the first oncoproteins from β-species that were shown to display transforming properties (Caldeira et al., 2003). Although many HPV variants have been described, HPV subtypes are not commonly reported. A subtype of HPV 38 with 96% L1 sequence similarity to reference HPV 38 was found in 2006 and called HPV 38b (subtype FA125) (Hazard et al., 2006). Among the 48 CC samples analyzed in this study, 7 were positive to HPV 38b subtype (8.33% attribution), showing that this is the subtype that is present in Mexican samples and that its prevalence is almost as high as the one of HPV 18.

Interestingly, Kazemian et al. sought to identify new virus-cancer associations by searching RNA sequencing data sets from more than 2,000 patients, encompassing 21 cancers from The Cancer Genome Atlas (TCGA), for the presence of viral sequences. Unexpectedly, they found sequences matching HPV 38 in 32 out of 168 (19%) RNA-Seq samples of uterine corpus endometrioid carcinoma (Kazemian et al., 2015), from those, authors detected that some sequences corresponded to HPV 38b. Despite authors mention that these data could be derived from a possible cross-contamination of the samples from The Cancer Genome Atlas Database, the fact that these samples were really infected with HPV 38 cannot be ruled out. In this way, HPV 38 could not only be infecting skin and cervix but could also be present in some other pathologies such as endometrial cancer. In addition, Hampras et al. analyzed 87 men samples from the HIM project and determined that HPV 38 was the most prevalent β*-PV* found in genital skin (32.2%) (Hampras et al., 2017).

Regarding HPV 80, we found that it ranks the 4th place in attribution (7.26%—Figure 3), even though there are very few studies that have reported the presence of this genotype. HPV 80 was isolated, full sequenced and characterized from histologically normal skin in 1998 by Delius et al. (Delius et al., 1998). Recently, its presence was detected in a study that aimed to know the nasal virome diversity from indigenous children at South American Villages (Altan et al., 2019). HPV 80 has also been observed in 10% of actinic keratosis samples (Nindl et al., 2007) and in a HIV-positive man from South Africa (Meiring et al., 2017).

On the other hand, as depicted in Figure 2, it is worth mentioning that only 11 out of the 51 genotypes detected (HPV 16, 18, 26, 33, 52, 53, 54, 59, 62, 70, and 81—all of them belonging to α-*PVs*) were amplified with both sets of primers (PGMY09/11 and FAP). However, concerning the HPVs 18, 52, and 62, FAP primers were more efficient, suggesting that there are some variants that can only be detected by FAP primers. In addition, 31 genotypes can be exclusively detected by using FAP primers (including 7 unclassified HPVs), while only 9 genotypes exclusively amplified with PGMY primers. It is noteworthy that some HPVs that are supposed to amplify with PGMY, amplified only with FAP, as in the case of HPVs 6, 11, and 42, meaning that the prevalence of these genotypes could be underestimated. Therefore, as many genotypes are only amplified and sequenced with PGMY or FAP primers, the use of both sets of primers is a good way to detect a broader range of HPV genotypes present in CC samples. Also, some sequences that amplified by NGS were not included in this study because they showed an identity inferior to 80% to any HPV reference genotype or unclassified HPV reported sequences, suggesting that they could be new HPV genotypes not described to date.

According to the International Agency for Research on Cancer (IARC), the α*-PVs* genus harbors the oncogenic genotypes associated to anogenital cancers: 16, 18, 31, 33, 35, 39, 45, 51, 52, 56, 58, and 59; HPV 68 has been classified as “probably carcinogenic to humans” and HPVs 5, 8, 26, 30, 34, 53, 66, 67, 69, 70 73, 82, 85, and 97 as “possibly carcinogenic to humans”; however, there is still not enough evidence to determine the carcinogenicity of β- and γ- *PVs* (excluding HPVs 5 and 8) (IARC Working Group on the Evaluation of Carcinogenic Risks to Humans, 2012). Cuzick and Wheeler, after analyzing international studies on CC with Roche's Cobas HPV test, externalized the need for expanding HPV genotyping and they proposed screening tests with a separate assessment for HPVs 16, 18, 31, and 33, a pool of hr-HPVs including 35, 45, 51, 52, and 58 and a pool of intermediate risk HPV types for 39, 56, 59, and 68 (Cuzick and Wheeler, 2016). Nonetheless, these proposed pools would not be the best combination for the Mexican population, since in the present study, the HPVs with a higher attribution (based on PGMY primers) were: 16 (49.8%), 18 (11.0%), 33 (6.1%), 59 (4.9%), 52/68 (3.7%), 66 (3.2%), and 39 (3.0%). Surprisingly, hr-HPVs 31, 56, and 58 were not present in any of the CC samples, and HPVs 35, 45, and 51 were rare, since each of them was only found in one patient. It is valuable to mention that, considering exclusively the NGS data, only the following HPVs were found as single infections: 16 (*n* = 7), 18 (*n* = 2), 33 (*n* = 1), 59 (*n* = 2), and 68 (*n* = 1) (see Table 1, NGS data); all the other genotypes were detected as coinfections. On the other side, HPV attribution (based on FAP primers) was found as follows: 16 (20.1%), 38b (8.3%), 80 (7.3%), 18 (6.0%), 52 (5.6%), 62 (5.0%), 107 (3.4%), and 5 (2.6%), among others. It is noteworthy that by NGS detection, HPV 80 was found as single infection in two cases and that HPV 107 was found in one case coinfected with the unclassified-HPVs FA39 and mSK_220. So, we propose to also include HPVs 5, 38/38b, 80, and 107 in the future HPV tests. Interestingly, these genotypes belong to the species β-2 and β*-*1 (Figure 4). Other genotypes found with an attribution higher than commonly described hr-HPVs were mSK_120, mSK_136, FA39, 122, and FA116, most of them probably also belong to β-2 and β-1 species. An important observation was the fact that with the *Linear Array HPV genotyping test*, we detected genotypes that were not found by NGS. This could most likely be due to cross-hybridization, as has already been observed with some HPVs (Artaza-Irigaray et al., 2019).

By comparing four HPV genotyping methods, Nilyanimit et al. conclude that although NGS is expensive and complex, it detects multiple HPVs with high sensitivity and propose this method as an alternative for diagnostic HPV genotyping in certain situations (Nilyanimit et al., 2018). Derived from our studies, we also propose to use NGS (using at least PGMY and FAP primers) to determine the presence of a greater number of genotypes, including β- and γ- *PVs* in precursor cervical lesions and in CC, in order to determine the most prevalent genotypes in each geographical region.

By comparing the genotypes detected in CC with those determined in CIN1 samples, very promising observations were found. Firstly, it should be noted that in the CIN1 samples that were HPV negative with *Linear Array* (PGMY-HPV-neg group), HPV 58 was the most common genotype identified, being present in 63% of the samples analyzed. In contrast, this genotype was not found in any CC sample. The fact that PGMY amplification-based tests could not detect this genotype may be due to the variability reported for this genotype in Mexico and in some regions of the world (Raiol et al., 2009; Yue et al., 2013; Chen et al., 2016; Conde-Ferraez et al., 2017). HPVs 30, 214, and 11 were also common in this group. In a study carried out in 109 cervical specimens from South African HIV-positive women, HPV 30 was present in 14.6% of the samples (Meiring et al., 2012); on the other hand, HPV 214 (a γ*-PV*) has been recently reported to be present in penile swabs in South Africa (Murahwa et al., 2019).

Furthermore, another very interesting observation derived from our analysis is that some genotypes were exclusively found in the CC samples, such as: 5, 26, 38b, 39, 68, 107, 122, 135, FA39, FA116, mSK_120 and mSK_136 and mSK_221, among others. It should be noted that several of them belong to β2 and β1 species (as seen in Figure 4). β2 species have also been prevalent in men who have sex with men (Torres et al., 2015) and in head and neck squamous cell carcinoma (Agalliu et al., 2016; Sabol et al., 2016), The limited knowledge about the epidemiology of genital β- and γ- *PVs* infection makes it difficult to resolve their possible role as carcinogenetic co-factors increasing the effect of α*-PVs* at these anatomical sites.

The limitation of this study is that the number of cases should be increased, including CIN2 and CIN3 samples as well as the follow-up of patients with CIN1. Currently, we are recruiting more patients with cervical cancer and cervical precursor lesions to broaden our search for HPVs belonging to genera other than α-papillomaviruses.

## Conclusion

This study highlights the presence of a greater number of HPV genotypes than reported until now in cervical cancer, that belong not only to alpha-*PV* genus, but also to the beta and gamma genera, including HPV genotypes that have not been classified yet. In Mexico, many of them have even greater prevalence and attribution than many other HPVs reported as high risk, such as 38b, 80, 107, 5, mSK_120, mSK_136, FA39, 122, and FA116, among others.

It will be essential to develop more accurate and specific diagnostic methods that allow the detection of a greater number of HPV genotypes adapted to the different geographical regions. Additionally, it will be crucial to determine if those most prevalent genotypes have oncogenic properties and to unravel the contribution of different genera HPV coinfections to cancer.

## Data Availability Statement

The datasets generated for this study can be found in the NCBI BioProject repository with the following accession numbers: PRJNA506700 (for CC samples), PRJNA506685 (for CIN1 samples with multiple HPV infections), and PRJNA506459 (for PGMY-HPV-negative CIN1 samples).

## Ethics Statement

The studies involving human participants were reviewed and approved by Comisión Nacional de Investigación Científica, Instituto Mexicano del Seguro Social, Project No. F-CNIC-2014-35. The patients/participants provided their written informed consent to participate in this study.

## Author Contributions

JC-R, VV-R, YL-H, and PP-S were involved in patient interviews and cervical sample recruitment. VV-R, PP-S, and AM-P carried out the Linear Array HPV genotyping tests. MF-M and DO performed PCR assays and the NGS experiments. MF-M, IB, and AW did the data analyses. MM-S performed the histopathological diagnosis of all patients. CA-I wrote the first draft of the manuscript. AA-L and LJ-S designed the study, supervised all experiments and analyses, and completed the manuscript. All authors read, suggested modifications, and approved the final manuscript.

### Conflict of Interest

The authors declare that the research was conducted in the absence of any commercial or financial relationships that could be construed as a potential conflict of interest.

## Footnotes

^1^International Human Papillomavirus Reference Center: Karolinska Institutet. Available from: http://www.nordicehealth.se/hpvcenter/.

^2^Fast QC. *Babraham Bioinformatics*. Available from: https://www.bioinformatics.babraham.ac.uk/projects/fastqc/.

## Abbreviations

HPV: Human Papillomavirus
HR-HPV: High-Risk HPV
CC: cervical cancer
NGS: Next Generation Sequencing.
